# Differences in IDO1^+^ dendritic cells and soluble CTLA-4 are associated with differential clinical responses to methotrexate treatment in rheumatoid arthritis

**DOI:** 10.3389/fimmu.2024.1352251

**Published:** 2024-05-22

**Authors:** Anikó E. Malik, Drew Slauenwhite, Sarah M. McAlpine, John G. Hanly, Jean S. Marshall, Thomas B. Issekutz

**Affiliations:** ^1^ Department of Pediatrics, Faculty of Medicine, Dalhousie Unversity, Halifax, NS, Canada; ^2^ IWK Health Centre, Halifax, NS, Canada; ^3^ Division of Rheumatology, Faculty of Medicine, Dalhousie University, Halifax, NS, Canada; ^4^ Queen Elizabeth II Health Sciences Center, Halifax, NS, Canada; ^5^ Department of Microbiology & Immunology, Faculty of Medicine, Dalhousie University, Halifax, NS, Canada

**Keywords:** rheumatoid arthritis, dendritic cells, tolerogenic, methotrexate, therapy response, IDO1, CTLA-4

## Abstract

**Objective:**

Antigen-presenting dendritic cells (DCs) and monocytes play an essential role in rheumatoid arthritis (RA) pathogenesis, however, their tolerogenic potential remains unclear. Herein, the tolerogenic profiles of DCs are characterized in treatment-naïve RA patients to determine their role to inflammatory arthritis management.

**Methods:**

Thirty-six treatment-naïve RA patients were enrolled, of which 62% were non-responders to methotrexate (MTX) monotherapy based on disease activity score (DAS) after 6-months of therapy. DC and monocyte subset frequencies, activation (CD40, CD86, CD209 expression), and tolerogenic profile (intracellular indoleamine-2,3-dioxygenase [IDO1] and cytotoxic T lymphocyte antigen 4 [CTLA-4] expression) were examined in the baseline peripheral blood by multicolor flow-cytometry. Soluble CTLA-4 (sCTLA-4) levels in plasma were measured.

**Results:**

DC subsets were decreased in RA compared to healthy controls (HC), and the frequency of conventional DCs (cDC) inversely correlated with inflammatory markers and improvement in disease activity. CD141^+^ cDC1s were the major IDO1-expressing cells. IDO1^+^cDC1s were reduced in RA patients compared to HC. The baseline frequency of IDO1^+^cDC1s inversely correlated with improvement in disease activity. CTLA-4 expression in CD1c^+^ cDC2s and monocytes was lower in RA patients compared to HC. Moreover, MTX-responders had a significantly lower frequency of IDO1^+^cDC1 cells and higher level of sCTLA-4 in the plasma compared to MTX non-responders. There was a strong predictive association of low IDO1^+^cDC1 cells, low sCTLA-4 and non-response to MTX.

**Conclusions:**

Our findings reveal altered DC and monocytes immunophenotypes that are associated with RA pathology and treatment response. The frequencies of tolerogenic IDO1^+^cDC1s and the low level of sCTLA-4 are strongly associated with MTX non-responsiveness and therapeutic outcome. These results suggest that investigation of the association IDO1^+^cDC1 and sCTLA-4 with response to treatment may be more generalizable to other autoimmune diseases.

## Introduction

Rheumatoid arthritis (RA) is a complex systemic autoimmune disorder, characterized by chronic joint inflammation that leads to progressive destruction of the articular cartilage and other articular structures, with a wide variety of clinical manifestations ranging from mild symptoms to severe inflammation and rapid joint destruction (1). The complexity of RA is further exacerbated by the fact that individuals with similar clinical symptoms can have widely divergent underlying immunological, genetic, and molecular pathways (2). This variability is evidenced by the inconsistent responses to conventional treatments and the range of clinical outcomes observed (3). Both the American College of Rheumatology (ACR) and the European League Against Rheumatism (EULAR) endorse methotrexate (MTX) as the initial disease-modifying antirheumatic drug (DMARD) for treating RA (4, 5). However, nearly half of the RA patients who start MTX therapy discontinue it within a three-year period, primarily due to treatment ineffectiveness and intolerance (6). Given that early mitigation of inflammation correlates with improved long-term results, prompt initiation of effective treatment following diagnosis is crucial (7). The challenge lies in accurately predicting patient outcomes, partly because the mechanisms that MTX targets are not fully elucidated (8).

A key event in the propagation of synovial inflammation is the infiltration of immune cells such as monocytes and dendritic cells (DCs), which after entering the tissue undergo further activation and differentiation (9). Several potential candidate genes involved in the pathogenesis of autoimmune diseases have been identified using genome-wide association studies and highlight the importance of antigen-presenting cells (APC) (10). DCs are important in initiating and orchestrating the immune response (11). They can also induce tolerance and regulate immune homeostasis (12). In human blood, cells with DC properties have been classified into conventional/classical DC (cDC) type 1 (CD141^+^ cDC1), cDC type 2 (CD1^+^ cDC2), and plasmacytoid DCs (CD123^+^ pDCs) (11). DCs play important roles in autoimmune disorders and while some DC functions are altered similarly across autoimmune diseases, others are disease-specific (e.g., DCs contribution to RA pathogenesis through the RANK–RANKL signaling pathway) (13, 14). Recent studies have highlighted the complex role of DCs in RA, including their ability to regulate the proliferation and differentiation of T cells, their involvement in immune tolerance, and their varied functions depending on their phenotypic characteristics and maturation status (15).

Monocytes include three major subsets, termed classical (CD14^hi^CD16^−^), intermediate (CD14^hi^CD16^+^), and non-classical (CD14^+^CD16^hi^) (16). Monocytes are innate immune cells and pivotal in RA pathogenesis, as they not only secrete pro-inflammatory mediators like TNF and IL-1β but also infiltrate the inflamed synovium, differentiating into macrophages and ultimately contributing to osteoclastogenesis (17, 18).

Multiple immune regulatory mechanisms must function to maintain immunological homeostasis, ensuring an appropriate response to infections and avoiding autoimmune reactions (19). Indoleamine-2,3-dioxygenase 1 (IDO1) catalyzes the initial rate-limiting step in the degradation of tryptophan along the kynurenine pathway, and consequently produces immunosuppressive metabolites. IDO1 has been associated with RA pathogenesis in several studies (20, 21). However, intracellular IDO1 has not been thoroughly investigated in DCs and monocytes in RA. The importance of the checkpoint molecule CTLA-4 in RA is demonstrated through the effectiveness of the biologic abatacept (22), often attributed to abated T cell function. However, neither the presence nor the role of intracellular CTLA-4 in DCs and monocytes in RA patients have been investigated. Furthermore, the level of soluble CTLA-4 concentration in the plasma was not assessed in the context of MTX response.

Given the roles of DCs and monocytes in initiating and propagating inflammation in RA, understanding changes in their tolerogenic properties is imperative. To our knowledge, ours is the first study examining the immunoregulatory profiles of tolerogenic IDO1^+^ and CTLA-4^+^ DC and monocyte subsets and sCTLA-4 in treatment-naive RA patients. We hypothesized that DC and monocyte homeostasis is disrupted in RA, impairing immunoregulation by decreasing tolerogenic cell subsets. Elucidating alterations in these cells may provide insights into disease mechanisms and pave the way for new therapeutic targets aimed restoring peripheral tolerance.

## Materials and methods

### Enrollment of patients and sample selection

Patients with treatment-naïve RA who met the 2010 American College of Rheumatology (ACR)/European League Against Rheumatism (EULAR) classification criteria [23] were recruited between 2016 and 2019 from the Rheumatology Clinic at The Arthritis Centre of Nova Scotia, Queen Elizabeth II Health Sciences Centre in Halifax, Canada. Patients with moderate-to-high disease activity, defined as a Disease Activity Score (DAS) in 28 joints using the erythrocyte sedimentation rate (DAS28-ESR) of >3.2, and no previous exposure to glucocorticosteroids or DMARDs were included. Clinical parameters included disease duration prior to diagnosis, acute phase markers (C-reactive protein [CRP], erythrocyte-sedimentation rate [ESR]), and autoimmune serostatus (rheumatoid factor [RF], autoantibodies to citrullinated protein antigens [ACPA]). Healthy volunteers of similar age and sex with no current infections or systemic autoimmune diseases were included as healthy controls (HCs). The study was approved by the Nova Scotia Health Authority Research Ethics Board. All participants provided written informed consent.

### Processing peripheral blood and synovial fluid samples

Heparinized venous blood was collected from all patients at study entry and 6 months after the initiation of MTX treatment. Blood was diluted with an equal volume of PBS pH 7.4 (Gibco). The diluted blood was layered on 15ml Ficoll-Paque Plus (GE Healthcare) and centrifuged at 900g for 20 min. After plasma collection, the peripheral blood mononuclear cell (PBMC) layer was collected, washed three times, resuspended in RPMI-1640 containing 10% dimethyl sulfoxide and 50% fetal bovine serum (FBS; Gibco), and frozen in liquid nitrogen. (See details of reagents in **Supplementary Table 1**)

Synovial fluid (SF) was collected from inflamed knee joints of two long-standing RA patients. Cell-free SF was separated, bulk SF cells were washed twice in PBS. SF mononuclear cells (SFMNCs) were purified by layering over 15ml Ficoll-Paque Plus and centrifuged at 540g for 25 min. SFMNCs were washed three times and stored in liquid nitrogen. (See details of reagents in **Supplementary Table 1**)

### Stimulation of PBMC

After thawing, 1 × 10^6^ PBMC were incubated in RPMI-1640 medium containing 10% FBS, 50 U/mL penicillin (Gibco), 50 µg/mL streptomycin (Gibco), 2 mM L-glutamine (Gibco), and 50 μM 2-mercaptoethanol (Gibco). The cells were activated in 12-well plates with 25 μg/mL polyinosinic:polycytidylic acid (poly(I:C); Invivogen) at 37°C in 5% CO_2_ for 5 hours. (See details of reagents in **Supplementary Table 1**)

### Immunophenotyping

Immunophenotyping of APCs in the peripheral blood and in the SF was performed using fluorochrome conjugated monoclonal antibodies (**Supplementary Table 2**). Fixation/permeabilization of the cells and intracellular staining were performed using the FoxP3/Transcription Factor Staining Buffer Set in accordance with the manufacturer’s instructions (eBioscience). Acquisitions were performed on a FACSymphony A5 flow cytometer (BD Biosciences). Data analysis was performed using FlowJo software (version 10; BD).

Cell analysis consisted of multistage gating (see **Supplementary Figure 1**). Monocyte subsets (CD3^–^CD56^–^CD19^–^) were identified as classical (CD14^hi^CD16^–^), intermediate (CD14^hi^CD16^hi^), and non-classical monocytes (CD14^–^CD16^hi^). DC subsets (CD3^–^CD56^–^CD19^–^CD14^–^CD16^–^HLA^-^DR^hi^) were further defined by expression of CD11c^+^CD141^+^ (cDC1), CD11c^+^CD1c^+^ (cDC2), and CD123^+^ (pDC). The frequency of surface activation markers CD40, CD86, and CD209, and intracellular IDO1 and CTLA-4 were assessed on DC and monocyte subsets (**Supplementary Figure 2**).

### Measurement of plasma sCTLA-4

The concentration of sCTLA-4 in plasma was measured by ELISA (R&D Systems Inc.) according to the manufacturer’s protocol. Each sample was diluted 1 : 2 and tested in duplicate. The lowest sensitivity threshold was 22 pg/ml.

### Statistical analysis

All statistical analyses were based on normality assessment using a Shapiro-Wilk normality test. Data sets were presumed to be non-normally distributed when normality could not be confirmed. Data from normal distributions are reported as mean ± standard error of the mean (SEM) or standard deviation (SD), whereas data from non-normal distributions are displayed as the median with interquartile range (25th and 75th percentile).

When the distribution was normal, the means were compared using an unpaired t-test with Welch’s correction. The Student’s paired t-test was used to compare paired samples. In the case of a non-Gaussian distribution, the Mann-Whitney test was used to compare the ranks of two groups, while the Wilcoxon matched-pairs signed-rank test was used to compare the means of paired samples. Correlation coefficients were calculated using the Spearman rank correlation test and the Pearson correlation test, where appropriate.

To evaluate diagnostic accuracy of the frequency of IDO1^+^ cDC1 and the plasma concentration of sCTLA-4 in methotrexate response, the area under the curve (AUC) in the receiver operating characteristic (ROC) curve was constructed. Cut-off values for the optimal frequency of the IDO1^+^ cDC1 subset and the plasma concentration of sCTLA-4 that would maximize the sensitivity and specificity of the AUCs were determined based on the maximal Youden’s index.

Statistical analyses were performed using GraphPad Prism version 9.3.1 for Windows (GraphPad Software, San Diego, California, USA). P values less than 0.05 were considered statistically significant.

## Results

### Characteristics of enrolled patients

The study included an RA cohort of 36 DMARD-naïve patients with recent-onset RA. After six months of MTX treatment, 14 patients (39%) were in remission or had low disease activity (based on their DAS28-ESR score (≤ 3.2; MTX responder), while in the other 22 patients (61%), moderate-to-high disease activity persisted (> 3.2; MTX non-responder). The mean age and gender distributions of the two groups were similar. There were no significant differences in baseline DAS28-ESR, CRP level, ESR, or ACPA or RF serostatus (**Table 1**).

**Table 1:** Characteristics of enrolled patients at baseline.

	HC	RA total	RA MTX responders	RA MTX non-responders
	(n=18)	(n=36)	(n=14)	(n=22)
**Age, mean ± SD years**	51.6 ± 20.9	61.1 ± 11.2	59.9 ± 11.6	61.7 ± 11.2
**Sex, no. female/male**	10/8	22/14	8/6	14/8
**Symptom duration, median (IQR) months**	-	5.5 (3–12)	4.5 (3–8.3)	5.5 (4–16)
**CRP, median (IQR) mg/L**	-	13.3 (6.3–26.3)	10.6 (6.3–19.9)	15.6 (6.5–46.5)
**ESR, median (IQR) mm/hour**	-	27 (10–46)	18 (10–36)	35 (11–51)
**DAS28-ESR, mean ± SD**	-	5.6 ± 1.2	5.1 ± 1.1	5.9 ± 1.2
**ACPA, no. positive/negative**	-	23/13	8/6	15/7
**RF, no. positive/negative**	-	25/11	10/4	15/7

*HC, healthy controls; RA, rheumatoid arthritis; MTX, methotrexate; CRP, C reactive protein; IQR, interquartile range; ESR, erythrocyte sedimentation rate; DAS28-ESR, Disease Activity Score in 28 joints using ESR; ACPA, anti-citrullinated protein antibody; RF, rheumatoid factor.

### Decreased frequency of DC subsets and inverse correlation with acute phase parameters and disease activity at baseline in RA patients

**Figure 1:**
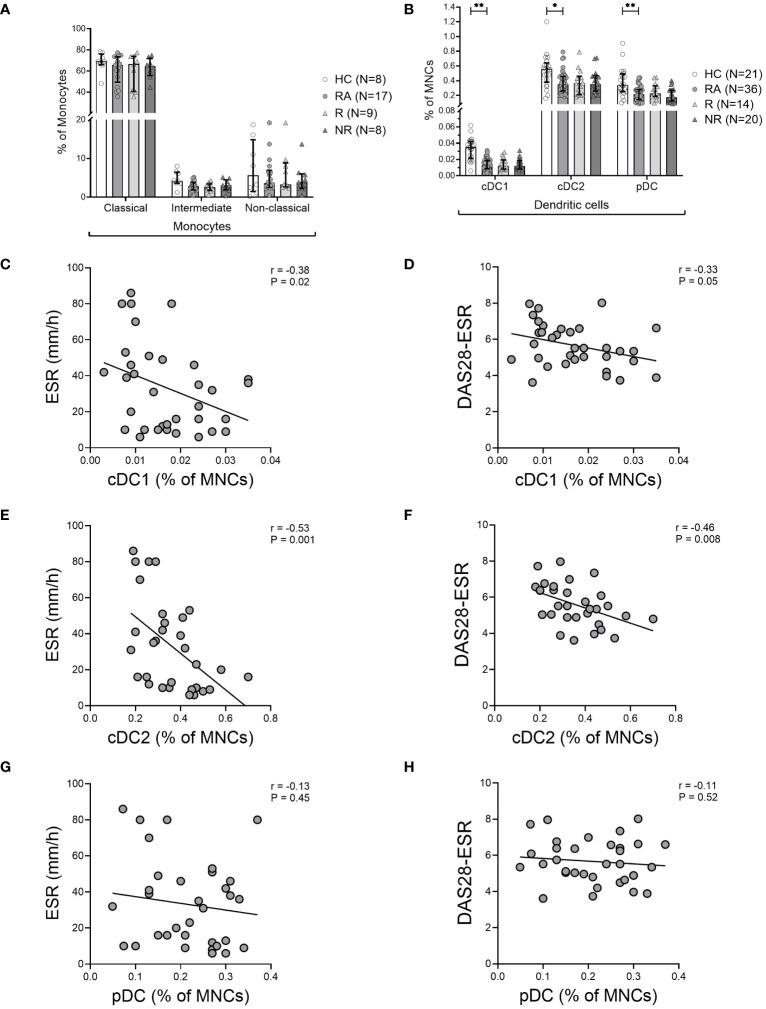
Decreased frequency of dendritic cells in the blood of RA patients. **(A)**, Frequency of monocyte subsets based on monocyte gate and **(B)**, Frequency of CD141^+^ cDC1, CD1c^+^ cDC2, and CD123^+^ pDC subsets based on the mononuclear cell gate (MNC) in the blood of healthy controls (HC), treatment-naïve rheumatoid arthritis (RA) patients, MTX-responders (R), and non-responders (NR) at baseline. Symbols represent individuals. Data are summarized using median and interquartile range. *=P < 0.05; **=P < 0.01; ***=P < 0.001; ****=P < 0.0001 by Mann-Whitney test. Correlations of DC subsets with erythrocyte sedimentation rate (ESR) **(C, E, G)**, and with disease activity measured by Disease Activity Score in 28 joints using erythrocyte sedimentation rate (DAS28-ESR) **(D, F, H)** at baseline. Associations between variables were analyzed by Spearman’s rank correlation test.

We quantified the three distinct DC subsets (cDC1 [CD141^+^], cDC2 [CD1c^+^], and pDC [CD123^+^]) and the three monocyte subsets (classical [CD14^hi^CD16^–^], intermediate [CD14^hi^CD16^hi^], and non-classical [CD14^–^CD16^hi^]) in the PBMC of HCs and treatment-naïve RA patients (see **Supplementary Figure 1**). The frequency of monocyte subsets in blood was comparable between our cohorts (**Figure 1A**). However, the frequency of all three DC subsets was significantly lower in the blood of RA patients (**Figure 1B**). There was no association between the frequency of any DC subset and the duration of symptoms prior to diagnosis or the improvement in disease activity following six months of MTX treatment (data not shown). Moreover, the frequency of DC subsets in MTX responders and non-responders was not significantly different at the time of the diagnosis (**Figure 1B**). However, the frequency of both cDC subtypes was inversely correlated with ESR and DAS28-ESR, but there was no correlation with CRP levels (**Figures 1C-F**). Similar to the cDCs, pDCs were also decreased in frequency in blood of RA patients, however, there was no correlation with ESR or DAS28-ESR (**Figures 1B, G, H**) suggesting that cDCs among the three types of DCs specifically correlate with DA, but pDCs do not.

Although SF samples were not available from the treatment naïve RA patients, we stained the SFMNC samples of two long-standing RA patients to investigate the frequency of DC subsets in the inflamed joint. We found that the frequency of both the cDC1s and cDC2s was increased in the SFMNC of the RA patients compared to matched PBMCs (**Supplementary Figure 3**). This suggests that the decreased cDCs in blood may relate to increased cDCs in the inflamed joint and therefore a negative correlation with disease activity as seen.

### CD209^+^ cDC2s are more abundant in the peripheral blood of RA patients

**Figure 2:**
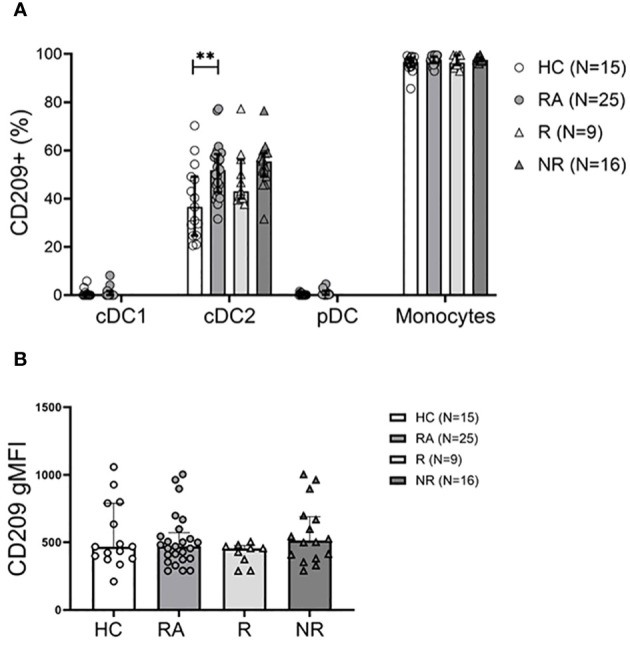
The CD209^+^ cDC2 subset is more abundant in RA patients. **(A)**, Frequency of the CD209^+^ DC subsets and CD14^+^ monocytes in healthy controls (HC), treatment naïve RA patients, MTX-responder (R) and non-responder (NR) patients at baseline. **(B)**, Geometric mean of surface CD209 expression on cDC2 cells. Data are summarized using median and interquartile range. **=P < 0.01 by Mann-Whitney test.

Next, we assessed activation markers (CD40, CD86, CD209) on the cell surface of the various DC subsets and discovered no difference in CD40^+^ and CD86^+^ subsets between RA and HC, or the MTX responders and non-responders (data not shown). CD209 (or DC-SIGN) is a marker of DC activation so its expression was determined (**Supplementary Figure 2B**). The frequency of CD209 on cDC2s, but not cDC1 or pDCs was increased in the RA patients (**Figure 2A**). The gMFI value did not differ between the groups (**Figure 2B**). The frequency of CD209^+^ cDC2s showed a correlation with serum CRP (r=0.41; P=0.04), but not with other clinical parameters or therapy response (data not shown).

### The frequency of IDO1^+^ cDC1 may predict MTX therapy response in RA

**Figure 3:**
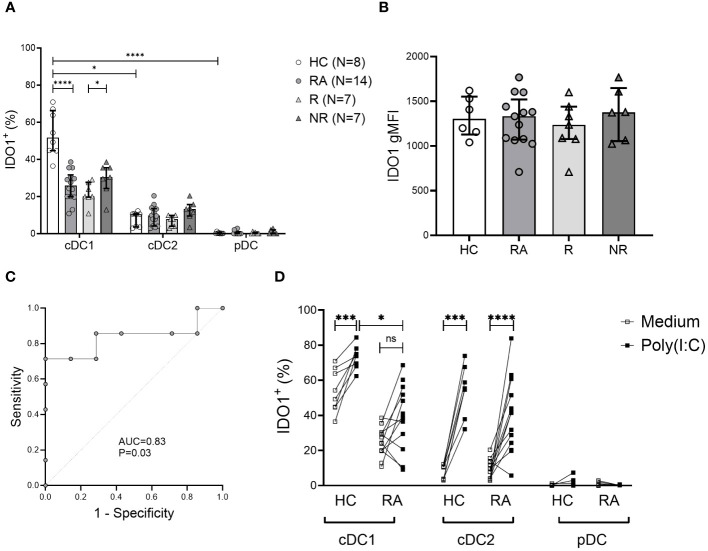
Frequency of tolerogenic IDO1^+^ cDC1 cells is decreased in RA and may be associated with MTX therapy response. **(A)**, Intracellular indoleamine-2,3-deoxygenase (IDO1) expression in DC subsets. Data are summarized using median and interquartile range. *=P < 0.05; **=P < 0.01; ***=P < 0.001; ****=P < 0.0001 **(B)**, Geometric mean of intracellular IDO1 expression on cDC1 cells. Data are summarized using median and interquartile range. **(C)**, Receiver operating characteristic curve showing area under the curve (AUC) for the frequency of IDO1^+^ cDC1 subset applied to distinguish MTX non-responders from responders. **(D)**, Intracellular IDO1 expression in DC subsets following 5h incubation with poly (I:C) vs. medium. Data are the individual matched values (connecting line) in 7–14 individuals. *=P < 0.05; **=P < 0.01; ***=P < 0.001; ****=P < 0.0001 by Wilcoxon matched-pairs signed rank test or Mann-Whitney test, based on normality.

We took the novel approach of evaluating the intracellular expression of the tolerogenic IDO1 and CTLA-4 molecules in DCs and monocytes in the context of RA (**Supplementary Figure 2A, C-E**). Among DC subsets, cDC1s were the major IDO1-expressing cells. RA patients had significantly lower frequency of IDO1^+^ cDC1 cells compared to HC (P<0.0001). Low IDO1^+^ cDC2 frequencies were detected and pDCs did not express intracellular IDO1 (**Figure 3A**). In addition, the frequency of IDO1^+^ cDC1s was significantly lower (P=0.03) in MTX responders compared to non-responders at baseline (**Figure 3A**). The gMFI values of intracellular IDO1 did not differ between the groups (**Figure 3B**).

We also measured the frequency of IDO1^+^ cDC1 cells in RA patients six months after the initiation of MTX treatment. There was no significant difference in the frequency of IDO1^+^ cDC1 cells between MTX responders and non-responders, (mean 25.56% ± 8.33 vs 31.26% ± 30.24, respectively). There was also no difference between samples from baseline or after six months treatment with MTX.

To assess the discriminative power of baseline IDO1^+^ cDC1 frequency regarding MTX response, we analyzed the area under the curve (AUC) in a receiver operating characteristic (ROC) curve (**Figure 3C**). The frequency of IDO1^+^ cDC1 had an AUC of 0.83 (P=0.03). Based on the coordinates of the ROC curve, the optimal cut-off value for IDO1^+^ cDC1 frequency in distinguishing MTX non-responders from responders was determined to be 28.4%. This cut-off value was associated with good sensitivity (71%) and a good positive predictive value (71%), while the specificity was 85%, and the negative predictive value was 71%.

The capacity of cDC1s to upregulate IDO1 in response to stimulation with the Toll-like receptor 3 (TLR3) agonist poly (I:C) was also reduced in RA compared to HC (**Figure 3D**). In contrast, cDC2s from both RA patients and HC exhibited equal ability to upregulate IDO1 following activation (**Figure 3D**). IDO1 upregulation in DC subsets did not differ between responders and non-responders (data not shown).

### The frequency of intracellular CTLA-4^+^ myeloid cells is decreased in RA patients

**Figure 4:**
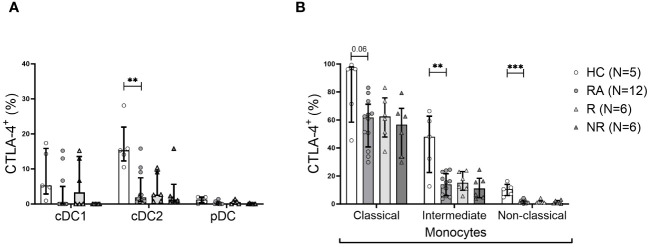
The frequency of CTLA-4^+^ cDC2 cells and monocyte subsets is lower in RA. **(A)**, Frequency of intracellular CTLA-4^+^ cells among DC subsets. **(B)**, Frequency of intracellular CTLA-4^+^ cells among indicated monocyte subsets. Data are summarized using median and interquartile range. **=P < 0.01; ***=P < 0.001 by Mann-Whitney test.

Intracellular CTLA-4 was present in cDC2s and in all the three monocyte subsets (**Figure 4**). Classical monocytes were the main CTLA-4^+^ subset, with the intermediate and non-classical subsets expressing this marker to a lesser extent. Importantly, the intracellular CTLA-4^+^ cDC2 and all three monocyte subsets showed lower frequency of intracellular CTLA-4^+^ phenotype (significantly lower in intermediate and non-classical monocytes) in RA patients compared to HC (**Figure 4**). However, there was no association with clinical parameters or therapy response (data not shown).

### Soluble CTLA-4 is increased in MTX-responders at baseline

**Figure 5:**
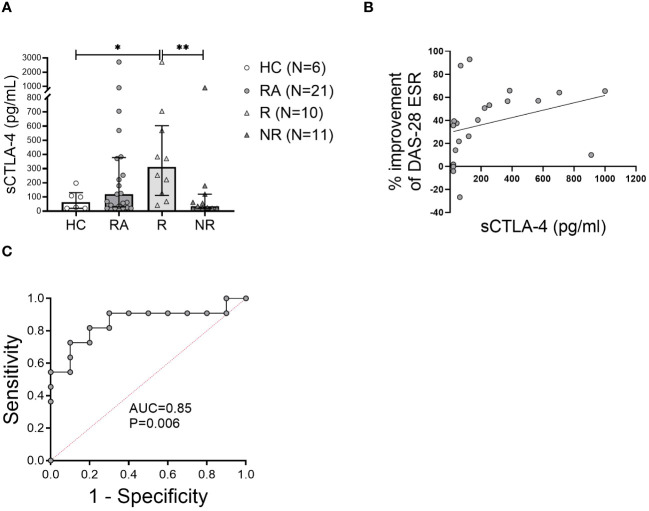
The level of soluble CTLA-4 in the plasma may predict MTX response at baseline. **(A)**, Soluble CTLA-4 (sCTLA-4) concentration was measured in the plasma of healthy controls (HC) and rheumatoid arthritis (RA) patients at baseline. Data are summarized as median and interquartile range. *=P < 0.05, **=P < 0.01 by Mann-Whitney test. **(B)**, Correlation of the plasma sCTLA-4 concentration with the improvement of DAS28-ESR disease activity index after 6 months of methotrexate treatment. Associations between variables were analyzed by Spearman’s rank correlation test. **(C)**, Receiver operating characteristic curve showing area under the curve (AUC) for the concentration of sCTLA-4 applied to distinguish MTX non-responders from responders.

sCTLA-4 in the plasma of RA patients and healthy controls was measured. Patients with RA at baseline had a higher mean sCTLA-4 level than HC (**Figure 5A**). Furthermore, MTX-responders had significantly (p<0.005) higher (9-fold) levels of sCTLA-4 compared to MTX-non-responders that were comparable to the levels in the HC (**Figure 5A**). The concentration of sCTLA-4 also showed a strong positive correlation with the improvement of the DAS28-ESR score after 6 months MTX therapy (**Figure 5B**).

We also measured sCTLA-4 levels in the plasma of RA patients six months after the initiation of MTX treatment; however, we found no significant difference between baseline and six month in either responders or non-responders. However, the difference between responders and non-responders observed at six months was similar to that at baseline (8-fold), namely responders were significantly higher than non-responders (median 175 pg/ml [IQR 678.8] vs. 20 pg/ml [154.4], p=0.034, respectively).

To determine the ability of baseline measures of sCTLA-4 to distinguish MTX responders from non-responders, the AUC in ROC curves was examined. The plasma concentration of sCTLA-4 had an AUC of 0.85 (*P* = 0.006) (**Figure 5C**). Based on the coordinates of the ROC curves, the optimal cutoff for the concentration of sCTLA-4 was 123 pg/ml. This cutoff value for the sCTLA-4 concentration was associated with good sensitivity (81%) and good positive predictive value (70%), while the specificity of this measure was 80% and the negative predictive value was 82%.

## Discussion

In this study we determined the activation and the tolerogenic profiles of DCs and monocytes in the blood of treatment naïve patients with RA and observed several novel findings. Our data demonstrated distinct immunological differences between RA patients and HCs, including decreased frequencies of all DC subsets, an increase of CD209^+^ cDC2s, and a lower frequency of tolerogenic cells including IDO1^+^ cDC1s, CTLA-4^+^ cDC2s and monocytes, respectively. In addition, sCTLA-4, which has a major role in tolerance, was increased in patients with RA. Importantly, our findings are the first to demonstrate that the RA patients who respond to treatment with MTX as first line therapy have a significantly lower frequency of IDO1^+^ cDC1s and a greatly increased level of plasma sCTLA-4 compared to MTX non-responders. In addition, these differences are substantial enough to be predictive of response or nor-response to MTX based on the ROC curves.

The decreased DC subsets in the blood of treatment naïve RA patients, is consistent with previously published data (9, 23). Furthermore, the frequencies of both cDC1 and cDC2 subsets in our cohort exhibited a significant negative correlation with ESR and disease activity at baseline Previous studies showed that the frequency of circulating cDC2s was inversely correlated to CRP, ESR, and disease activity in RA patients at baseline, but cCD1s had not been examined (23). The observed inverse associations between clinical inflammatory parameters (such as ESR) and DC subsets may be explained in part by inflammatory cytokines (such as IL-6 and TNF), which play a major role in RA pathophysiology and whose serum levels frequently correlate with disease activity (24).

In comparison to the findings in the blood, a large accumulation of cDCs and pDCs in the synovial fluid of inflamed joints of long-standing RA patients was observed. An interesting possible explanation linking these findings with previous observations is demonstrated by alterations in the chemotactic profiles of DCs in the blood of RA patients (9, 23, 25). Once in the joint, decreased emigration of DCs from this inflamed site to draining lymph nodes further contributes to their accumulation (13). Alternatively, or perhaps additionally, decreased frequency of DCs in the blood may imply altered production, lifespan, or phenotypic plasticity in RA (13).

We demonstrated, for the first time, that the frequency of CD209^+^ cDC2s is increased in RA patients compared to HC. The increased CD209^+^ cDC2s represent an important finding given the role of CD209 in autoimmunity and cartilage destruction in RA (26). CD209 is a C‐type lectin expressed on DCs and binds ICAM-3, establishing and stabilizing contact between DCs and resting T cells (27). CD209, found here to be increased on cDC2s in the blood of RA patients, also mediates DC trafficking through interactions with endothelial ICAM-2 (28, 29), that may contribute to the decreased frequency of cDC2s in the blood. Marzaioli et al. demonstrated in RA patients, that peripheral CD209^+^ DCs express more inflammatory cytokines (IL-1β/IL-6/IL-12/TNFα) compared to HC (30). *CD209* promoter polymorphisms have been associated with increased susceptibility to autoimmunity, including RA (26, 31) and higher expression of CD209 on immune cells correlated with the heightened severity of cartilage destruction in patients with RA (26). Taken together with our findings, increased frequency of CD209^+^ cDC2 cells is intriguing as an important aspect of the immune dysregulation in RA and warrants further investigation.

We found that IDO1^+^ tolerogenic cDC1s were significantly decreased in RA patients, especially in MTX responders. IDO1 expression by DCs converts mature DCs towards a tolerogenic phenotype that suppress T_effector_ cell and enhance T_regulatory_ cell activity, thereby promoting tolerance (32). Proposed mechanisms for the immunosuppressive activity associated with IDO1 are through local tryptophan depletion and the generation of immune suppressive tryptophan catabolites, which both contribute to tolerogenic processes by activating metabolic pathways responsive to amino acid withdrawal and aryl hydrocarbon signaling, respectively (33).

Under homeostatic conditions, IDO1 is selectively expressed by cDC1s (34). IFNγ signaling or TLR ligation further increases IDO1 expression in cDC1s and induces modest expression in cDC2s, but not in pDCs (34). Indeed, we found that in HC and RA, cDC1s were the main IDO1 expressing DC subset in the blood. Comparatively, IDO1^+^ cDC2s were less abundant and IDO1 expression was not detected among pDCs. Moreover, the frequency of IDO1^+^ cDC1s was lower in RA compared to HC, largely attributable to MTX responders among our cohort. IDO1 has been shown to have a protective effect within inflamed joints in preclinical models of RA (21, 35), through suppression of T_effector_ cell secretion of the proinflammatory cytokines IFNγ and IL-17 (36). However, inhibition of IDO has also been shown in the KBxN mouse model of RA to ameliorate arthritis by decreasing autoreactive B cell response (37).

Stimulation of TLR3 and TLR4 induces IDO1 and CTLA-4 in DCs (38). IDO1 activity in TLR-stimulated DCs limits T cell proliferation by depleting tryptophan and enriching its inhibitory metabolites (34). Therefore, we hypothesized there is a difference between RA patients and HC in their ability to upregulate IDO1 in response to TLR signaling. Indeed, after poly (I:C) stimulation of PBMC, RA patients showed a lower capacity for IDO1 upregulation in cDC1s compared to HC. The reason for this difference is not clear, however, epigenetic modifications of the IDO1 gene may be involved (39) or there maybe polymorphisms in genes regulating IDO-1 expression.

Although CTLA-4 is thought to be expressed nearly exclusively by lymphoid cells, previous studies have shown that mature myeloid or cDCs express high levels of intracellular CTLA-4, which they constitutively secrete in microvesicular structures (40). These CTLA-4^+^ microvesicles competitively bind B7 costimulatory molecules (CD80/CD86) on bystander DCs, resulting in their downregulation (40). We and others observed intracellular CTLA-4 in cDC2s, with lower levels detected in cDC1s (38). Interestingly, the frequency of intracellular CTLA-4^+^ cCD2s was decreased in the blood of RA patients compared to HC. Furthermore, CTLA-4^+^ classical and intermediate monocytes, were also abundantly present in the healthy controls, while their frequency was lower in RA patients, implying that the less tolerogenic profile of these cells might contribute to chronic inflammation in RA. The lower frequencies of monocytes and cDC2 cells expressing intracellular CTLA-4 in RA patients compared to HC indicates dysregulation in the expression or shedding of CTLA-4 from monocytes in RA.

Monocytes are known to secrete CTLA-4, contributing to the levels of soluble CTLA-4 in the circulation (41). sCTLA-4 is an immune-checkpoint molecule that is significantly elevated in RA patients compared to controls (42). The levels of sCTLA-4 can vary depending on the stage of the disease and the treatment status, with higher levels observed in untreated RA patients (43).We investigated for the first time how the levels of sCTLA-4 in RA patients were associated with MTX response. Our finding that sCTLA-4 levels might predict MTX response is particularly intriguing. However, it is important to note that these findings need to be validated in larger cohorts and across different populations to establish the robustness of the association between sCTLA-4 levels and MTX response. Additionally, the underlying mechanisms by which sCTLA-4 levels may influence or reflect the response to MTX treatment should be further explored. If confirmed, sCTLA-4 could potentially serve as a predictive biomarker to identify RA patients who are more likely to respond favorably to MTX treatment and more importantly those who are unlikely to respond. Further research into the function of DC and monocyte-produced CTLA-4 is crucial, particularly considering the therapeutic efficacy of the biologic CTLA-4 fusion protein abatacept (44) Our results also suggest that there may be mechanistic immunologic differences between the patients who have low IDO1^+^ cDC1s and high sCTLA-4. This suggests that RA may be able to be subdivided into two groups based on these tolerogenic differences at the time of presentation.

Although patients with RA often share similar clinical features at diagnosis, including joint pain, swelling, and stiffness, their response to therapy can be highly variable (45). This heterogeneity underscores the complex nature of RA, where seemingly identical clinical pictures can mask diverse underlying mechanisms and disease trajectories (2). The predictive value of baseline IDO1^+^ cDC1 frequencies and the baseline sCTLA-4 plasma levels in MTX response suggests these tolerogenic cells and molecules could enable personalized medicine approaches upon validation in a larger RA cohort. Patients unlikely to respond to MTX could be identified early and offered alternative therapies such as biologics. The role of tolerogenic DC-based therapies represents a new approach to treat autoimmune diseases by rebalancing the immune system (46) and may offer a safer (minimal adverse effects compared to conventional immunosuppressive treatment) and a more effective way to promote peripheral tolerance (47). The generation of tolerogenic DCs - as an autologous cellular therapy - involves the isolation of DC precursors from the patient, their differentiation *ex vivo* into tolerogenic DCs, and their subsequent injection back into the patient. Active research is being conducted on the generation and function of tolerogenic DCs in relation to autoimmune and autoinflammatory diseases (48).

The limitations of this study include the small sample size. Larger cohorts are needed to confirm our findings and longitudinal assessment will provide deeper insights.

## Conclusions

This study provides several novel findings that significantly advance our understanding of DCs and monocytes in RA. We report decreased tolerogenic IDO1-expressing cDC1s and CTLA-4-expressing cDC2s and monocytes in RA patients. These findings indicate an impaired immunoregulatory capacity among APCs, which may promote autoimmunity. Furthermore, we present evidence that the frequency of IDO1+ cDC1s and the plasma concentration of sCTLA-4 in RA patients prior to treatment has predictive value in distinguishing MTX responders from non-responders. Once validated, these novel biomarkers could further inform treatment decisions. Overall, our findings reveal immunoregulatory changes in DCs and monocytes that may contribute to RA pathology and treatment responses. In addition, these findings point to new potential therapeutic targets.

## Data availability statement

The raw data supporting the conclusions of this article will be made available by the authors, without undue reservation.

## Ethics statement

The studies involving humans were approved by Nova Scotia Health Authority and IWK Health Centre. The studies were conducted in accordance with the local legislation and institutional requirements. The participants provided their written informed consent to participate in this study.

## Author contributions

AM: Conceptualization, Formal analysis, Funding acquisition, Investigation, Methodology, Project administration, Visualization, Writing – original draft, Data curation, Writing – review & editing. DS: Methodology, Writing – review & editing. SM: Conceptualization, Methodology, Writing – review & editing, Formal analysis. JH: Conceptualization, Investigation, Writing – review & editing, Data curation. JM: Conceptualization, Supervision, Writing – review & editing, Funding acquisition. TI: Conceptualization, Formal analysis, Funding acquisition, Methodology, Supervision, Writing – review & editing, Resources.

## Supplementary material

Supplementary Figure 1 Gating strategy for identification of monocyte and dendritic cell subsets by multicolor flow cytometry. In the first stage leukocytes were gated on forward scatter (FSC) and side scatter (SSC). Single cell discrimination was then performed by side scatter height (SSC-H) and side scatter area (SSC-A). Next, the dead cells and NK cells were excluded using Fixable Viability Stain 575V and CD56, respectively. After exclusion of CD3^+^ T cells and CD19^+^ B cells from gated mononuclear cells (MNCs), the monocytes and dendritic cells were identified in the CD19^-^CD3^-^ cell population. In the next stage the three monocyte subsets were determined based on their CD14 and CD16 expression (CD14hiCD16^-^ classical monocytes; CD14hiCD16hi intermediate monocytes; CD14^-^CD16hi non-classical monocytes). The HLA-DR^+^ cells in the CD14-CD16- gate were further divided based on the expression of CD123^+^ (pDC), CD11c^+^CD141^+^ (cDC1) and CD11c^+^CD1c^+^ (cDC2). The two last panels in the 2nd row display the same CD11c^+^ gate with pseudocolor vs. density plot.

Supplementary Figure 2 FACS gating of intracellular IDO1, CTLA-4 and surface CD209. Representative histograms showing the gating strategy for selected markers based on Fluorescence Minus One Controls (represented by grey shading). **(A)**, Intracellular IDO1 on cDC1 cells. **(B)**, Surface CD209 on cDC2 cells. **(C-E)**, Intracellular CD152 = CTLA-4 on Classical, Intermediate and Non-Classical Monocytes.

Supplementary Figure 3 cDC subsets are more abundant at the site of inflammation. Representative flow cytometry dot plots showing the frequency of CD1c^+^ cDC1 and CD141^+^ cDC2 cells within total CD11c^+^ cDCs from **(A)**, peripheral blood mononuclear cells (PBMC) and **(B)**, synovial fluid mononuclear cells (SFMNC) from an individual RA patient’s samples.
