# Supplementary figures and images for: Differences in IDO1+ dendritic cells and soluble CTLA-4 are associated with differential clinical responses to methotrexate treatment in rheumatoid arthritis
Source: Front Immunol. 2024 May 22;15:1352251. doi: 10.3389/fimmu.2024.1352251 (PMC11150726; doi:10.3389/fimmu.2024.1352251)

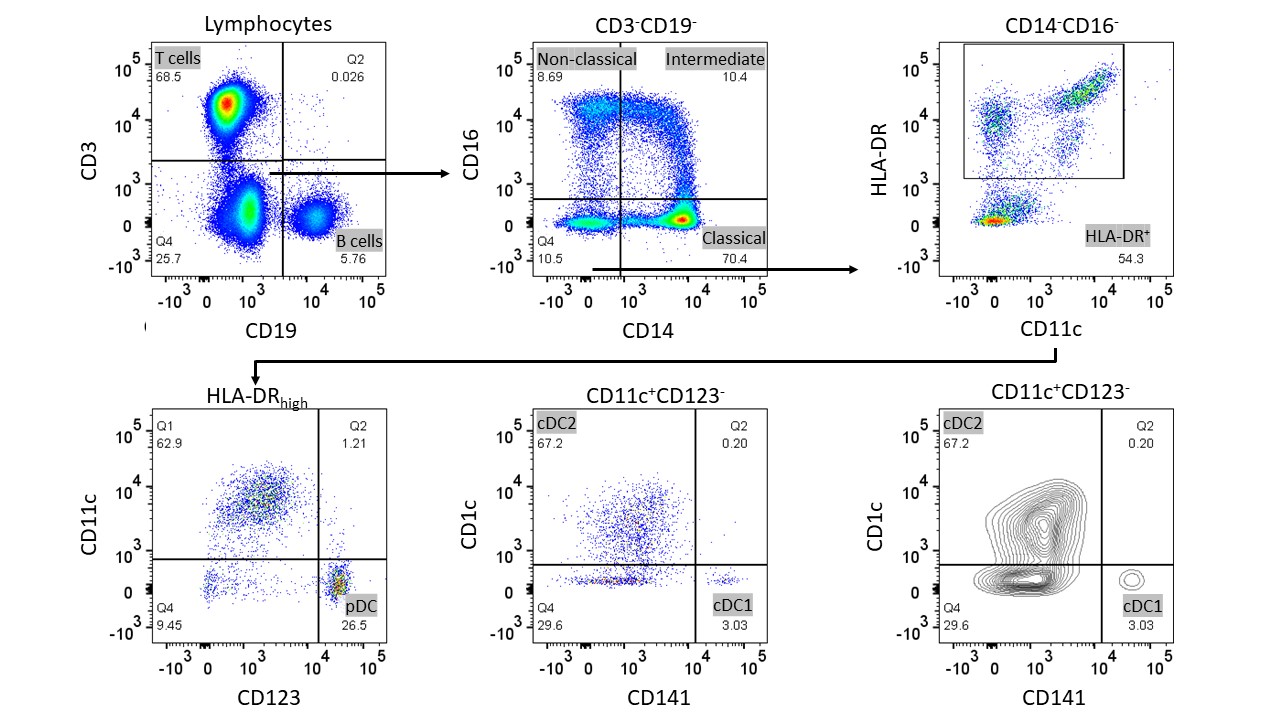

Supplement: Supplementary Figure 1 — Gating strategy for identification of monocyte and dendritic cell subsets by multicolor flow cytometry. In the first stage leukocytes were gated on forward scatter (FSC) and side scatter (SSC). Single cell discrimination was then performed by side scatter height (SSC-H) and side scatter area (SSC-A). Next, the dead cells and NK cells were excluded using Fixable Viability Stain 575V and CD56, respectively. After exclusion of CD3+ T cells and CD19+ B cells from gated mononuclear cells (MNCs), the monocytes and dendritic cells were identified in the CD19-CD3- cell population. In the next stage the three monocyte subsets were determined based on their CD14 and CD16 expression (CD14hiCD16- classical monocytes; CD14hiCD16hi intermediate monocytes; CD14-CD16hi non-classical monocytes). The HLA-DR+ cells in the CD14-CD16- gate were further divided based on the expression of CD123+ (pDC), CD11c+CD141+ (cDC1) and CD11c+CD1c+ (cDC2). The two last panels in the 2nd row display the same CD11c+ gate with pseudocolor vs. density plot. [file Image_1.jpeg]

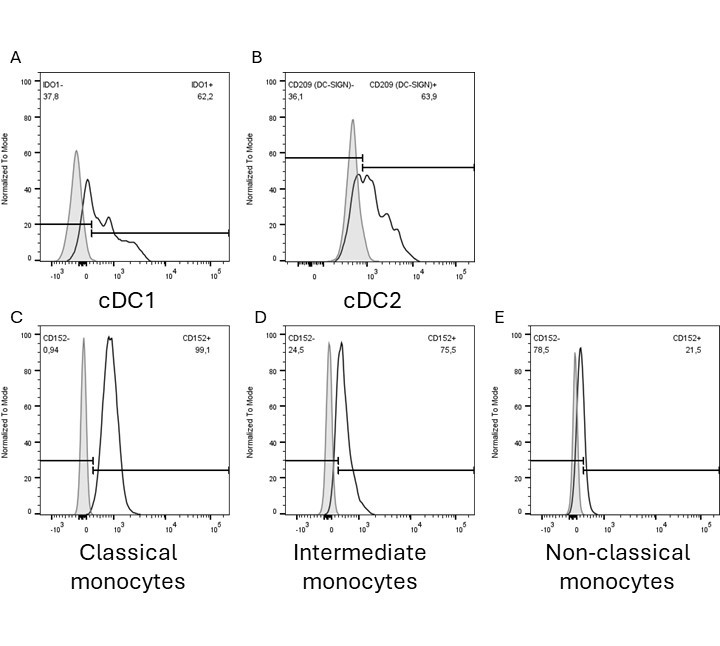

Supplement: Supplementary Figure 2 — FACS gating of intracellular IDO1, CTLA-4 and surface CD209. Representative histograms showing the gating strategy for selected markers based on Fluorescence Minus One Controls (represented by grey shading). (A), Intracellular IDO1 on cDC1 cells. (B), Surface CD209 on cDC2 cells. (C-E), Intracellular CD152 = CTLA-4 on Classical, Intermediate and Non-Classical Monocytes. [file Image_2.jpeg]

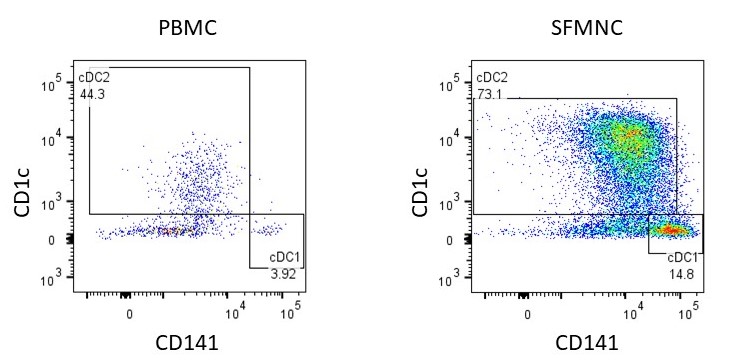

Supplement: Supplementary Figure 3 — cDC subsets are more abundant at the site of inflammation. Representative flow cytometry dot plots showing the frequency of CD1c+ cDC1 and CD141+ cDC2 cells within total CD11c+ cDCs from (A), peripheral blood mononuclear cells (PBMC) and (B), synovial fluid mononuclear cells (SFMNC) from an individual RA patient’s samples. [file Image_3.jpeg]
