# Supplementary material for: Differences in IDO1+ dendritic cells and soluble CTLA-4 are associated with differential clinical responses to methotrexate treatment in rheumatoid arthritis
Source: Front Immunol. 2024 May 22;15:1352251. doi: 10.3389/fimmu.2024.1352251 (PMC11150726; doi:10.3389/fimmu.2024.1352251)
Supplement: Supplementary file 4 [file Table_1.pdf]

Supplementary Table 1 Reagents used for peripheral blood and synovial fluid processing and for peripheral blood stimulation.

**Reagent name**	**Company**	**Catalogue number**
PBS pH 7.4	Thermo Fisher / Gibco	10010023
Ficoll-Paque Plus	GE Healthcare	17144003
Fetal Bovine Serum	Thermo Fisher / Gibco	16000044
RPMI-1640	Thermo Fisher / Gibco	11875093
Dimethyl sulfoxide	Thermo Fisher / Gibco	D12345
Penicillin-Streptomycin	Thermo Fisher / Gibco	P4333
L-glutamine	Thermo Fisher / Gibco	25030149
2-mercaptoethanol	Thermo Fisher / Gibco	31350010
Polyinosinic:polycytidylic acid (HMW)	Invivogen	Tlrl-pic
