# Supplementary material for: Differences in IDO1+ dendritic cells and soluble CTLA-4 are associated with differential clinical responses to methotrexate treatment in rheumatoid arthritis
Source: Front Immunol. 2024 May 22;15:1352251. doi: 10.3389/fimmu.2024.1352251 (PMC11150726; doi:10.3389/fimmu.2024.1352251)
Supplement: Supplementary file 5 [file Table_2.pdf]

Supplementary Table 2 Antibodies and reagents used for immunofluorescent staining.

**Marker**	**Clone**	**Fluorochrome**	**Supplier**
CD1c	F10/21A3	BB515	BD Biosciences
CD3	SK7	BV786	BD Biosciences
CD4	SK3<br>M-T477	BUV805<br>BV510	BD Biosciences
CD11c	B-ly6<br>3.9	BV650<br>BV785	BD Biosciences<br>BioLegend
CD14	M5E2	BV605	BD Biosciences
CD16	3G8	APC-H7	BD Biosciences
CD19	HIB19	BUV496<br>PE-Cy7	BD Biosciences
CD40	5C3	BUV737<br>APC-H7	BD Biosciences
CD56	5.1H11	BV570	BioLegend
CD86	IT2.2	PE	BD Biosciences
CD123	7G3	PE-Cy7<br>BV421	BD Biosciences
CD141	1A4	BV711	BD Biosciences
CD152 (CTLA-4)	BNI3	BV421	BD Biosciences
CD209	9E9A8	FITC	BioLegend
HLA-DR	G46-6	BUV395<br>AF700	BD Biosciences
IDO1	V50-1886	AF647	BD Biosciences
Viability Dye	-	FVS575V	BD Biosciences
